# Machine learning models to predict pathological complete response after chemoradiotherapy in muscle-invasive bladder cancer: toward optimized indications for bladder preservation

**DOI:** 10.1016/j.ctro.2026.101320

**Published:** 2026-10-13

**Authors:** Motohiro Fujiwara, Hajime Tanaka, Daisuke Hirahara, Tatsunori Saho, Eichi Takaya, Shunya Matsumoto, Kasumi Yoshitomi, Masaki Kobayashi, Yuki Nakamura, Wei Chen, Yudai Ishikawa, Shohei Fukuda, Hiroshi Fukushima, Yuma Waseda, Soichiro Yoshida, Yasuhisa Fujii

**Affiliations:** a Department of Urology, Institute of Science Tokyo, Tokyo, Japan; b Department of Medical Course, Faculty of Health and Medical Science, Teikyo Heisei University, Tokyo, Japan; c Department of Radiological Technology, Kokura Memorial Hospital, Fukuoka, Japan; d AI Laboratory, Tohoku University Hospital, Miyagi, Japan; e Graduate School of Information Sciences, Teikyo Heisei University, Tokyo, Japan

**Keywords:** Muscle-invasive bladder cancer, Chemoradiotherapy, Machine Learning, Pathologic complete response, Radiotherapy

## Abstract

**Background and purpose:**

After bladder-sparing chemoradiotherapy (CRT) for muscle-invasive bladder cancer (MIBC), residual microscopic disease may cause intravesical recurrence. This study aimed to develop machine learning models to predict pathological complete response (pCR) after CRT.

**Materials and methods:**

We retrospectively analyzed 285 patients with cT2-4N0M0 MIBC who underwent CRT followed by partial or radical cystectomy. CRT response was determined from cystectomy specimens. Patients were randomly assigned to a training cohort (*n* = 228) and a test cohort (*n* = 57) using a fixed random seed. A total of 102 machine learning models were evaluated in the training cohort. The model with the highest area under the receiver operating characteristic curve (AUC) in the training-cohort was selected and then evaluated in the test cohort. The model incorporated clinical T stage, tumor size, multiplicity, hydronephrosis, bladder neck involvement (BNI), concomitant carcinoma in situ (CIS), variant histology, grade, and progression.

**Results:**

Clinical T stages were T2/3/4 in 169/105/11 patients, respectively. Hydronephrosis was observed in 69 patients. Transurethral resection before CRT revealed multiplicity, BNI, and concomitant CIS in 108, 54, and 34 patients, respectively. Overall, 178/285 patients (62%) achieved pCR. WeightedEnsemble_L4 was selected as the final model and had a test-cohort AUC of 0.829 (95% confidence interval [CI], 0.706–0.932). The calibration slope was 0.856 (95% CI, 0.469–1.639), and the wide CI indicated uncertainty in calibration.

**Conclusion:**

Machine learning based on clinicopathologic variables estimated pCR after CRT. This may support decision-making regarding bladder preservation in patients with MIBC.

## Highlights

• We studied 285 CRT-treated patients with muscle-invasive bladder cancer.
• Surgical pathology showed 62% pathological complete response after CRT.
• ≥cT3 stage, tumor size >30 mm, CIS, and multiple tumors predicted poor CRT response.
• Machine learning predicted CRT response with a test AUC of 0.83.

Phone: +81-3-5803-5295.

## 1 Introduction

Radical cystectomy has long been the standard treatment for muscle-invasive bladder cancer (MIBC), although it carries risks of perioperative complications and reduced quality of life related to urinary diversion. [1], [2] Multimodality bladder-preservation therapy based on chemoradiotherapy (CRT) is an alternative curative treatment. [3] Trimodality therapy, which consists of maximal transurethral resection of bladder tumor (TURBT) followed by CRT, is the most widely used bladder-preservation approach. [3] Previous studies have reported survival outcomes comparable to radical cystectomy in selected patients, and current guidelines include bladder preservation as a treatment option for appropriately selected patients. [4], [5], [6] However, primary resistance to CRT remains an important clinical problem. [7] Optimal patient selection remains difficult because most bladder-preservation studies include highly selected patients and usually assess CRT response with imaging and TURBT rather than complete surgical pathology. [8] Imaging and TURBT can miss microscopic residual cancer, which may later cause intravesical recurrence.

Since 1997, our institution has used a tetramodality bladder-preservation protocol that combines maximal TURBT, induction CRT, and partial cystectomy at the original tumor site. [9], [10], [11] We have also used CRT before radical cystectomy in patients who were not candidates for bladder preservation. [10], [11], [12] These treatment series provide surgical specimens after CRT and allow direct pathologic assessment of treatment response. We therefore analyzed patients with MIBC who underwent CRT followed by partial or radical cystectomy and developed models to estimate the probability of pCR (ypT0) after CRT. Machine learning allowed systematic evaluation of routinely available clinicopathologic variables, while surgical pathology was used to determine CRT response.

## 2 Materials and methods

### 2.1 Patients

This study included patients with MIBC who were enrolled in one of two treatment protocols at our institution: the tetramodality bladder-preservation protocol or presurgical CRT before radical cystectomy. Both protocols included CRT followed by partial or radical cystectomy. [9], [12] Written informed consent was obtained from all patients before protocol enrollment. After institutional review board approval (approval number: M2019–192), we retrospectively reviewed 314 patients with cT2-4N0M0 bladder cancer who had pathologically confirmed urothelial carcinoma and received either protocol between 1997 and 2021. Twenty-nine patients (9.2%) did not undergo partial or radical cystectomy because of distant metastasis (*n* = 6, 1.9%) or patient withdrawal (*n* = 23, 7.3%) and were excluded. The final study cohort included 285 patients (91%) who underwent partial cystectomy (*n* = 148) or radical cystectomy (*n* = 137) after CRT. Fig. 1 shows patient inclusion. No formal sample size calculation was performed; all eligible patients treated during the study period were included.

**Fig. 1:**
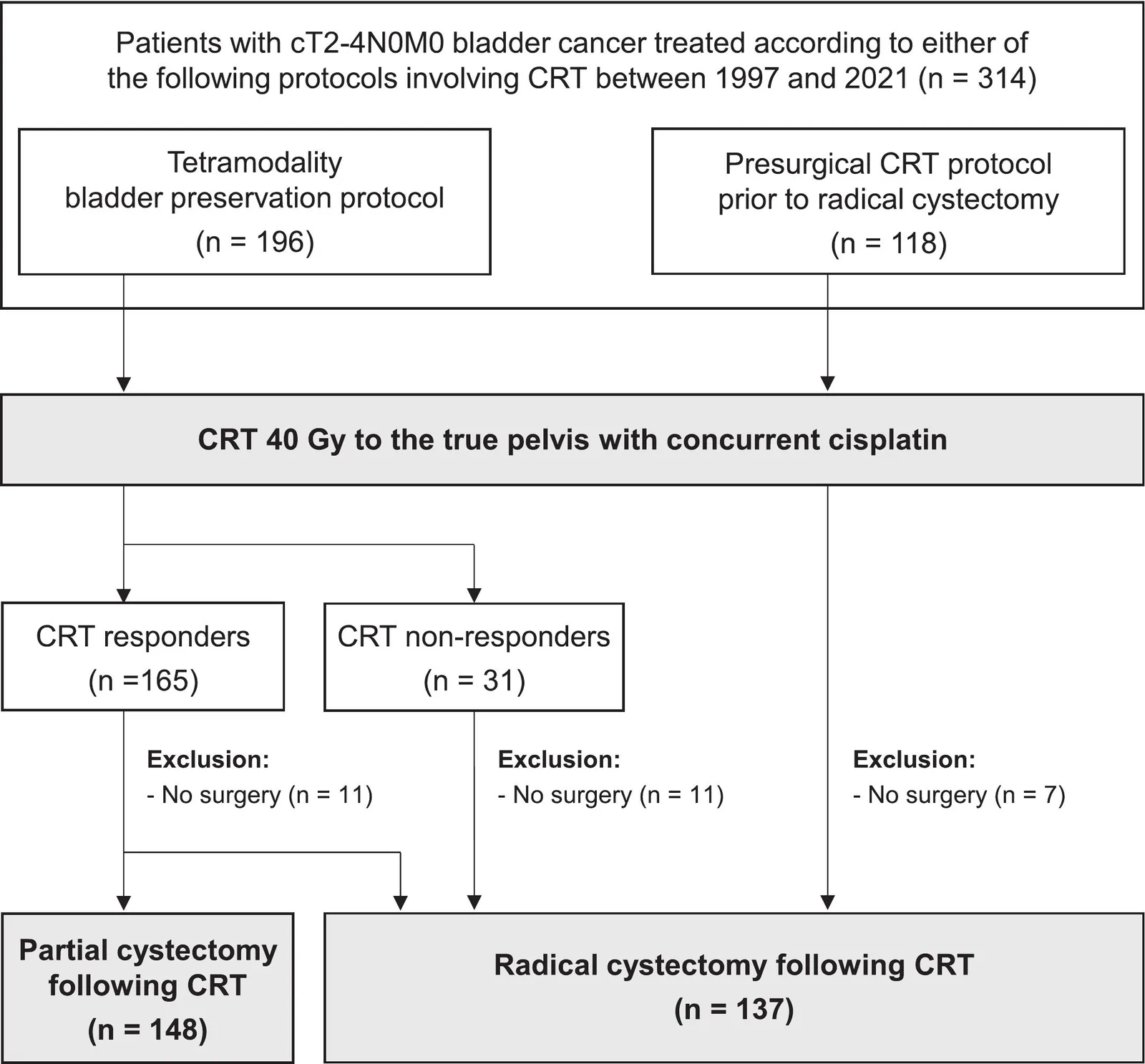
Flow diagram of study inclusion.

Patient and tumor characteristics and pathologic findings were obtained from medical records. Clinical stage was assigned according to the eighth edition of the TNM classification. [13] Staging included computed tomography of the chest, abdomen, and pelvis and magnetic resonance imaging of the bladder. Tumor size was defined as the maximum tumor diameter measured on pretreatment computed tomography or magnetic resonance imaging. For model development, tumor size was classified as >30 mm or ≤ 30 mm. Primary histologic grade was assessed in the pretreatment TURBT specimen using the 1999 WHO classification. Histologic variants and subtypes were assessed according to the 2016 WHO classification. [14], [15] Progression from prior non-muscle-invasive bladder cancer (NMIBC) was defined as recurrence as MIBC after prior pathologically confirmed NMIBC had been treated before the current CRT course.

### 2.2 Treatment protocols

The tetramodality bladder-preservation protocol consisted of maximal TURBT, induction CRT, and consolidative partial cystectomy. CRT consisted of external-beam radiotherapy of 40 Gy in 20 fractions to the true pelvis without boost radiation and two cycles of concurrent cisplatin (20 mg/day for 5 days) separated by a 3-week interval. The cisplatin dose was reduced for patients with poor performance status or impaired renal function. After CRT, response was assessed with imaging, urine cytology, and TURBT. Patients with a complete clinical response underwent consolidative partial cystectomy at the original tumor site. Radical cystectomy was offered to patients without a complete clinical response. Patients in the presurgical CRT protocol who were not candidates for bladder preservation received the same CRT regimen followed by radical cystectomy. Detailed eligibility criteria and treatment regimens have been reported previously. [9], [12]

### 2.3 Study endpoint and machine learning approach

The study endpoint was pCR (ypT0) after CRT, determined from partial or radical cystectomy specimens. We used AutoGluon, an open-source automated machine learning framework for tabular data. [16], [17], [18], [19] The following pretreatment clinicopathologic variables were included based on prior studies: clinical T stage (≥cT3), tumor size (>30 mm), bladder neck involvement (BNI), hydronephrosis, multiplicity, concomitant carcinoma in situ (CIS), primary histologic grade (G3), variant histology, and progression from prior NMIBC. [20], [21], [22], [23]

The 285 patients were randomly assigned to a training cohort (*n* = 228) and a test cohort (*n* = 57). The split was performed without stratification. Model training, model ranking, and final model selection were completed in the training cohort before test-cohort performance was examined. AutoGluon trained and evaluated 102 candidate models using bagging and multilayer stacking. The candidate models included simpler base-level models, including regularized logistic regression. Candidate models were ranked by AUC from internal validation within the training cohort, and the model with the highest AUC was selected.

After final model selection, the selected model was evaluated in the test cohort. Discrimination was assessed with AUC. Calibration was assessed with a calibration plot. Ninety-five percent CIs for test-cohort AUC and calibration slope were calculated with 2000 stratified bootstrap samples. Decision curve analysis was retained as an exploratory analysis for the selected final model.

To assess whether performance depended on a single patient split, we performed additional internal validation using five different train/test splits of the 285 patients. In each analysis, 228 patients were used for model development and 57 patients were reserved for evaluation. The same AutoGluon modeling procedure was repeated independently for each split, and the WeightedEnsemble_L4 generated in each analysis was evaluated in the corresponding test cohort. Details of the additional internal validation are provided in the Supplementary Document.

After final model selection and test-cohort performance assessment, an exploratory permutation analysis in the training cohort was used to rank the relative contribution of the nine predictors. This post hoc analysis was not used for model training or model selection. The highest-ranked clinical factors were then examined for their associations with pCR and survival outcomes.

### 2.4 Statistical analysis

Continuous and categorical variables were compared with the Mann-Whitney *U* test and Fisher exact test, respectively. Metastasis-free survival and overall survival (OS) were calculated from the date of partial or radical cystectomy. Survival was estimated with the Kaplan-Meier method and compared with the log-rank test. All analyses other than machine learning were performed with JMP Pro version 17.0 (SAS Institute Inc., Cary, NC, USA) and R version 4.0.2 (R Foundation for Statistical Computing, Vienna, Austria). All tests were two-sided, and *p* < 0.05 was considered significant. We followed the TRIPOD statement for prediction model development and internal validation (Supplementary Table 1). Machine learning analyses used AutoGluon Tabular version 1.3.1 with Python version 3.11.13. There were no missing data for the predictors or the outcome.

## 3 Results

### 3.1 Patient characteristics

Table 1 summarizes the baseline characteristics of the 285 patients. The median age was 69 years, and 212 patients (74%) were male. Clinical T stage was T2 in 169 patients (59%), T3 in 105 (37%), and T4 in 11 (4%). Median tumor size was 3.0 cm (interquartile range [IQR], 2.0–4.0 cm). BNI was present in 54 patients (19%). Pretreatment hydronephrosis was present in 69 patients (24%), 108 (38%) had multiple tumors, and 34 (12%) had concomitant CIS. All patients had urothelial carcinoma, and 60 (21%) had variant histology.

**Table 1:** Patient characteristics.

	**All patients** **(*n* = 285)**	**Training cohort (*n* = 228)**	**Test cohort** **(*n* = 57)**	***p* value**
Age, years	69 (62–74)	69 (61–74)	70 (62–75.3)	0.798
Sex: male	212 (74)	170 (75)	42 (74)	0.867
Clinical T stage				0.892
T2	169 (59)	137 (60)	32 (56)	
T3	105 (37)	82 (36)	23 (40)	
T4	11 (4)	9 (4)	2 (4)	
Tumor size, cm	3.0 (2.0–4.0)	3.0 (2.0–4.0)	2.8 (2.0–4.0)	0.732
Tumor location: BNI	54 (19)	45 (20)	9 (16)	0.574
Hydronephrosis	69 (24)	51 (22)	18 (32)	0.167
Multiplicity: multiple	108 (38)	85 (37)	23 (40)	0.760
Concomitant CIS⁎	34 (12)	27 (12)	7 (12)	1.000
Primary histological grade⁎				0.699
G2	50 (18)	39 (17)	11 (19)	
G3	235 (82)	189 (83)	46 (81)	
Variant histology⁎	60 (21)	47 (21)	13 (23)	0.718
Progression from prior NMIBC	27 (9)	24 (11)	3 (5)	0.314

Values are n (%) or median (IQR).

Abbreviations: BNI, bladder neck involvement; CIS, carcinoma in situ; IQR, interquartile range; NMIBC, non-muscle-invasive bladder cancer.

⁎ Pathological findings in the pretreatment transurethral resection specimen.

### 3.2 Machine learning model for predicting pathological response to chemoradiotherapy

Pathologic assessment of partial or radical cystectomy specimens showed pCR in 178/285 patients (62%). The training cohort included 228 patients, with pCR in 142 (62%). The test cohort included 57 patients, with pCR in 36 (63%). Supplementary Table 2 provides detailed pathologic findings from cystectomy specimens.

Among 102 models, WeightedEnsemble_L4 was selected because it had the highest training-cohort AUC (0.921). Training-cohort results for all candidate models are shown in Supplementary Table 3. In the test cohort, WeightedEnsemble_L4 had an AUC of 0.829 (95% CI, 0.706–0.932; Fig. 2). The calibration slope was 0.856 (95% CI, 0.469–1.639). The wide CI indicates uncertainty in calibration within the 57-patient test cohort. Calibration and exploratory decision curve results for WeightedEnsemble_L4 are shown in Supplementary Fig. 1. Additional internal validation results are provided in Supplementary Table 4.

**Fig. 2:**
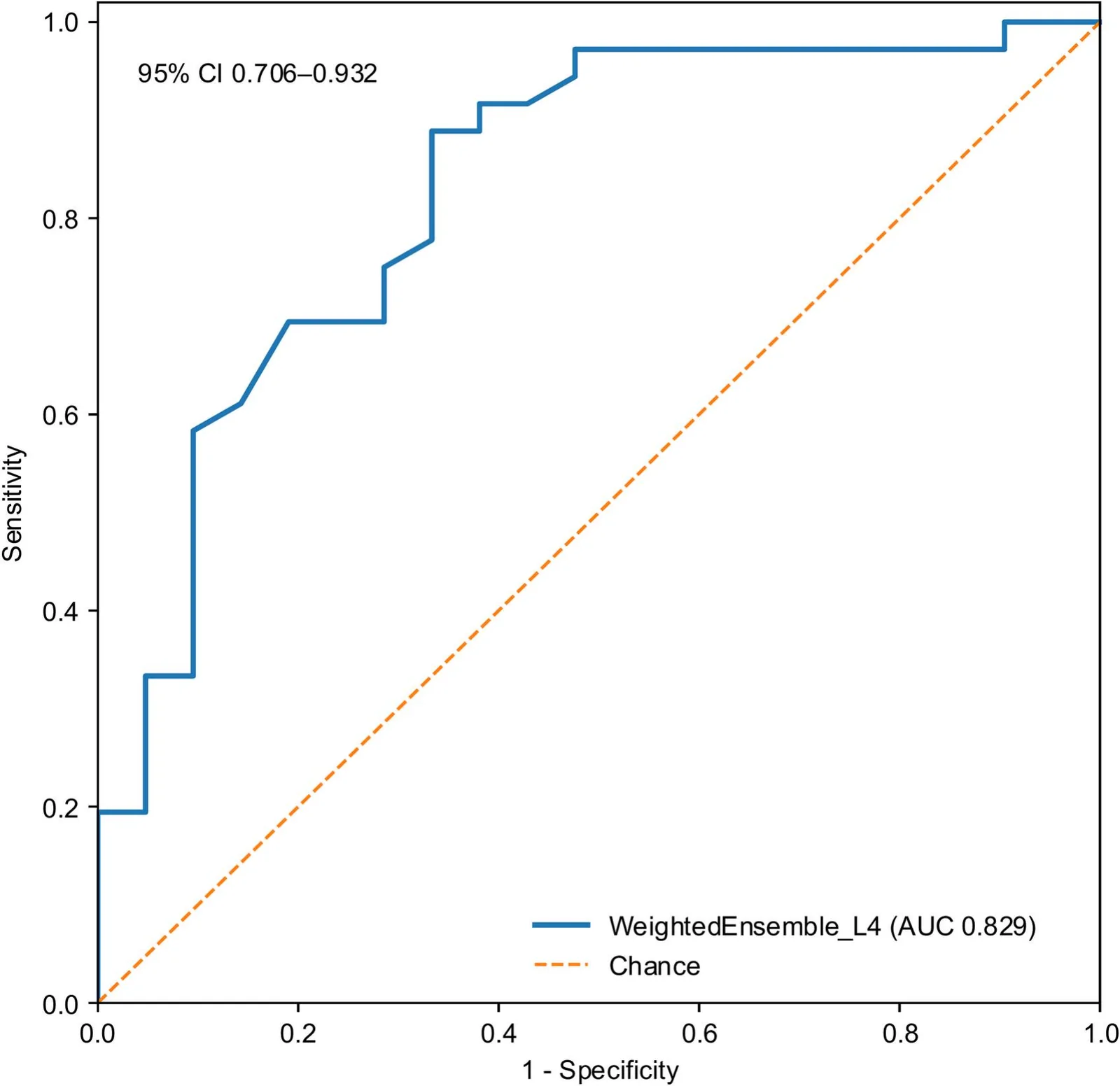
Receiver operating characteristic curve for WeightedEnsemble_L4 in the test cohort. The area under the receiver operating characteristic curve was 0.829 (95% confidence interval, 0.706–0.932).

### 3.3 Clinical factors associated with pathological complete response

The exploratory permutation analysis in the training cohort ranked ≥cT3, tumor size >30 mm, concomitant CIS, and multiplicity as the four leading clinical factors (Fig. 3). In the training cohort, pCR was less frequent in patients with these factors than in patients without them: ≥cT3, 46/91 (50.5%) versus 96/137 (70.1%); tumor size >30 mm, 37/77 (48.1%) versus 105/151 (69.5%); concomitant CIS, 13/27 (48.1%) versus 129/201 (64.2%); and multiplicity, 39/85 (45.9%) versus 103/143 (72.0%) (Fig. 4a).

**Fig. 3:**
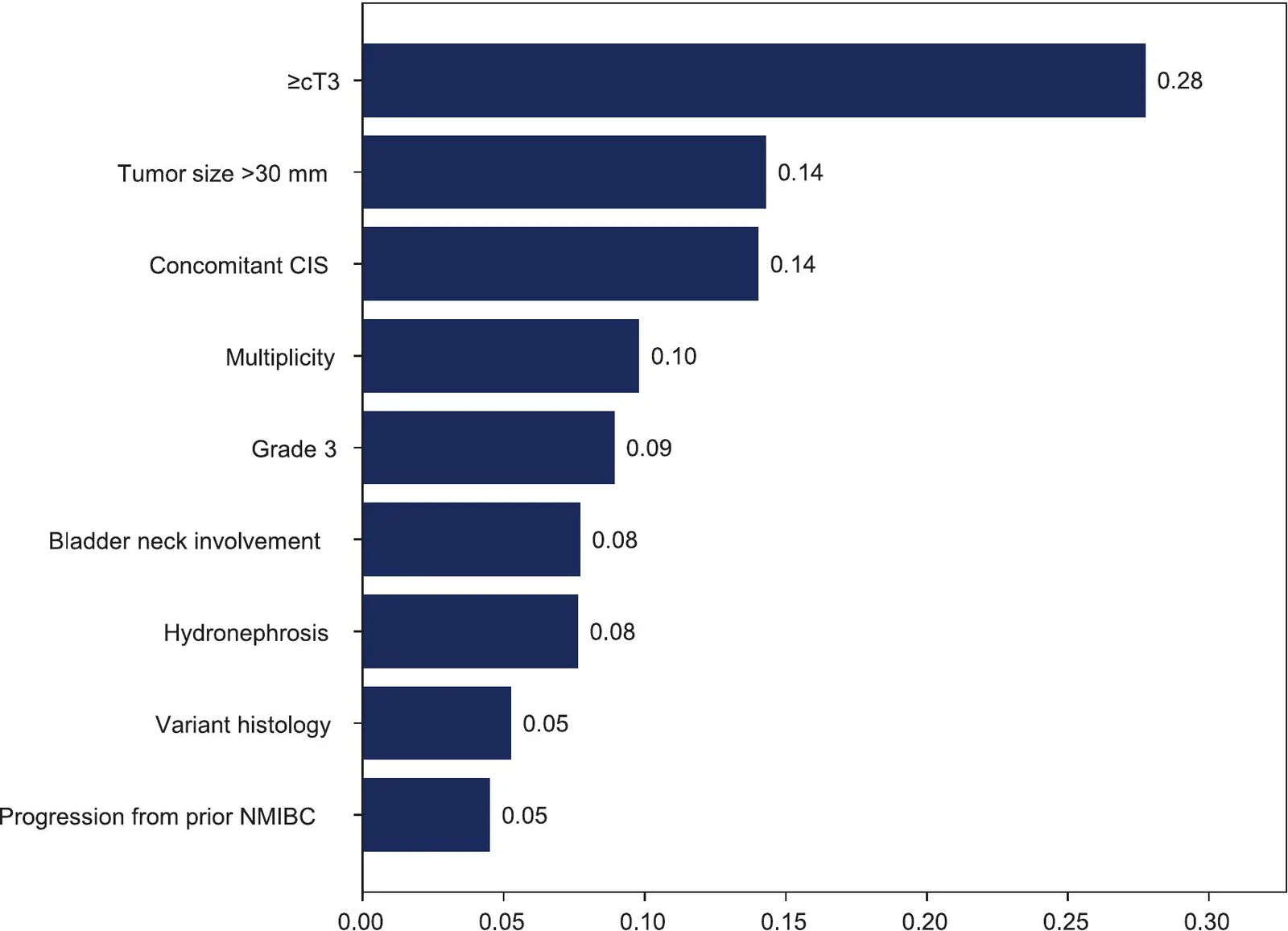
Exploratory permutation-based ranking of clinicopathologic variables in the training cohort after final model selection and test-cohort performance assessment. Results were averaged across eight models.

**Fig. 4:**
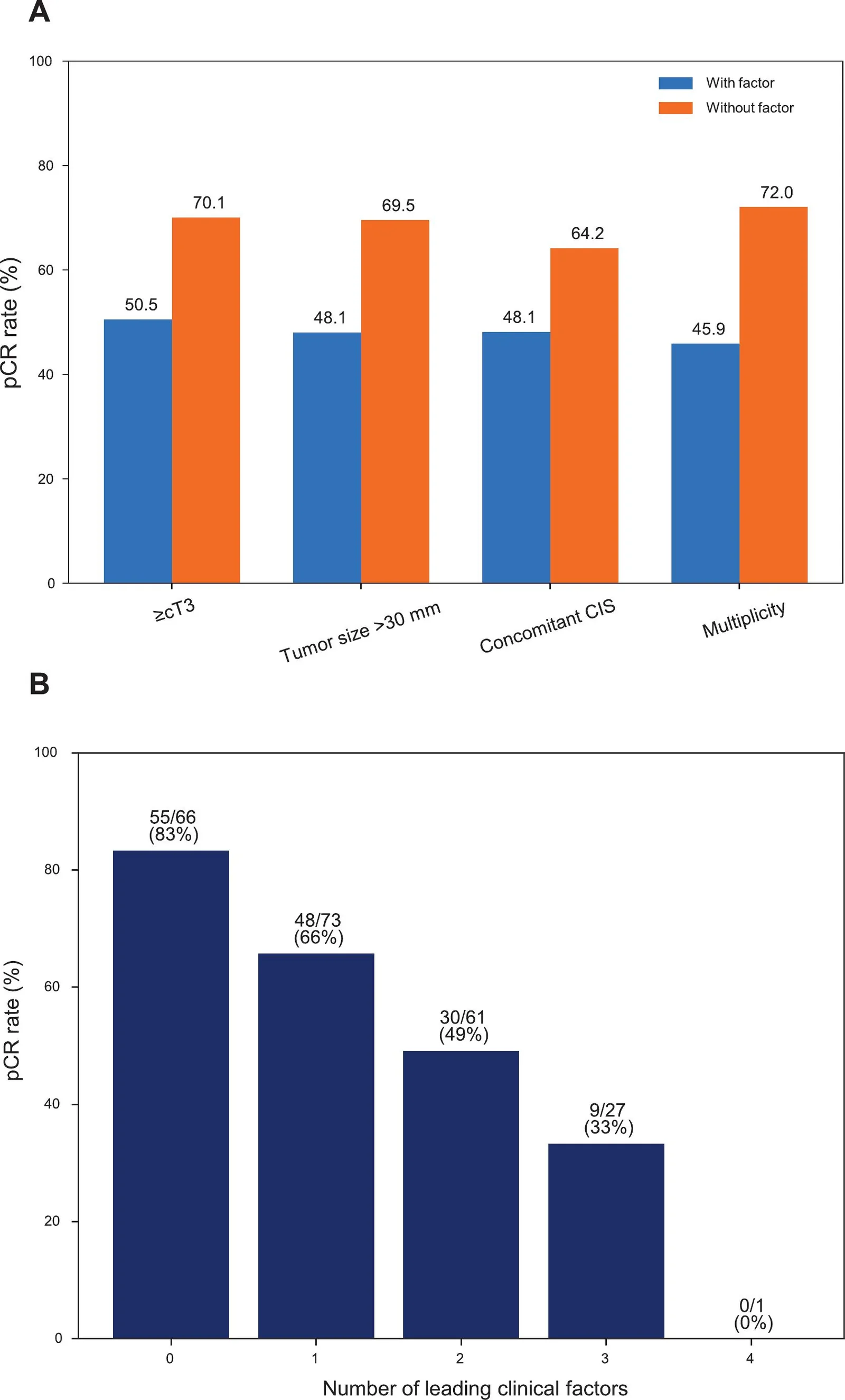
(a) Pathological complete response (pCR) rates according to each of the four leading clinicopathologic factors in the training cohort. (b) pCR rates according to the number of these four factors.

We also examined the number of these four factors as an exploratory clinical summary. In the training cohort, 66 (29%), 73 (32%), 61 (27%), 27 (12%), and 1 (0.4%) patients had 0, 1, 2, 3, and 4 factors, respectively. The corresponding pCR rates were 83%, 66%, 49%, 33%, and 0% (*p* < 0.001; Fig. 4b). Similar findings were observed in the full study cohort. The pCR rates were 87%, 66%, 49%, and 29% among patients with 0 (*n* = 84), 1 (*n* = 88), 2 (*n* = 72), and ≥ 3 (*n* = 41) factors, respectively (*p* < 0.001).

### 3.4 Survival outcomes after chemoradiotherapy followed by partial or radical cystectomy

During a median follow-up of 64 months, 58 patients (20%) developed metastatic recurrence and 70 patients (25%) died from any cause. The 5-year metastasis-free survival and overall survival rates were 79.0% and 79.7%, respectively. When patients were grouped by the number of the four clinical factors, the 5-year metastasis-free survival rates were 87.5%, 86.5%, 70.9%, and 59.8% for patients with 0 (*n* = 84), 1 (*n* = 88), 2 (*n* = 72), and ≥ 3 (*n* = 41) factors, respectively (*p* < 0.001; Fig. 5a). The corresponding 5-year overall survival rates were 86.9%, 85.1%, 72.8%, and 66.1% (*p* = 0.041; Fig. 5b).

**Fig. 5:**
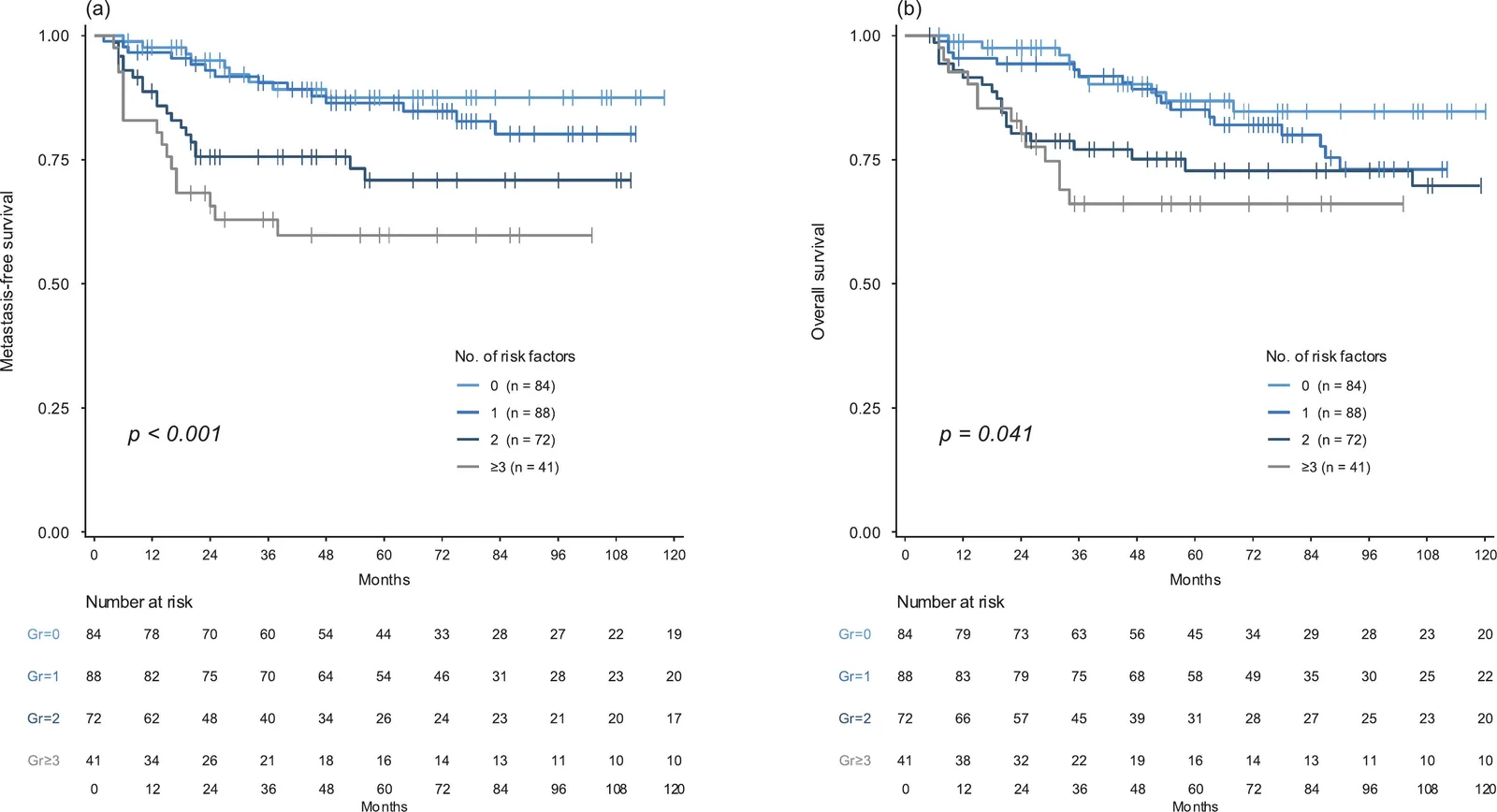
Survival outcomes after chemoradiotherapy followed by partial or radical cystectomy according to the number of the four leading clinicopathologic factors. (a) Metastasis-free survival. (b) Overall survival.

## 4 Discussion

Radical cystectomy remains the standard treatment for MIBC, while bladder-preservation therapy is an established option for selected patients. CRT response strongly affects whether bladder preservation succeeds. Several studies have examined clinical factors associated with CRT response, but most bladder-preservation cohorts include selected patients and do not provide complete surgical pathology after CRT. [24], [25], [26], [27]

The study has two clinical strengths. First, surgical pathology was used to define pCR after CRT. Second, the cohort included patients treated with a bladder-preservation protocol and patients treated with presurgical CRT before radical cystectomy. Including both groups may have reduced selection bias related to preselection for bladder preservation.

WeightedEnsemble_L4 had an AUC of 0.829 in the test cohort. Performance was lower in the additional five-fold internal validation, showing that the result varied across patient splits.

Previous studies used MRI-derived features and machine learning to predict CRT response in MIBC. [28], [29] AutoGluon allowed us to evaluate 102 candidate models using the same nine routinely available clinical variables, without radiomic feature extraction. We did not directly compare our approach with these MRI-based methods. We do not claim that the multilayer ensemble is superior to simpler models.

The exploratory analysis ranked ≥cT3, tumor size >30 mm, concomitant CIS, and multiplicity as the leading clinical factors associated with lower pCR rates. Advanced clinical stage and larger tumor size may reflect greater tumor burden and intratumoral heterogeneity. Concomitant CIS and multiplicity may indicate more extensive urothelial disease beyond the dominant tumor. Progression from prior NMIBC may reflect a more aggressive disease course and continued clonal evolution. Tuo et al. described clonal heterogeneity and clonal evolution as potential contributors to treatment resistance in bladder cancer. [30] Our study did not directly assess tumor genomics, the tumor microenvironment, or mechanisms of radiation resistance. These biological explanations therefore remain hypotheses that require direct testing.

The number of the four leading clinical factors was associated with pCR in both the training cohort and the full study cohort. The same clinical summary was also associated with metastasis-free and overall survival after surgery. These analyses were exploratory and were not used to train the machine learning model. Because all patients underwent partial or radical cystectomy after CRT, the survival results cannot be directly applied to patients managed with bladder preservation alone.

This study has several limitations. First, the retrospective, single-center design limits applicability to other institutions. External validation should use the final model in independent, preferably multi-institutional cohorts with different patient populations and CRT practices. Second, the training cohort was relatively small for evaluating many complex models. Model performance was lower when the analysis was repeated with different patient groups, suggesting that the results may depend on how patients were divided. Therefore, overfitting cannot be excluded. Third, selection bias may remain despite inclusion of patients with and without a preference for bladder preservation. Twenty-nine patients who did not undergo partial or radical cystectomy because of distant metastasis or withdrawal were excluded. Fourth, pretreatment imaging was not standardized, which may have affected clinical T staging and tumor-size measurement. Finally, the calibration slope in the test cohort had a wide 95% CI, so predicted pCR probabilities remain uncertain.

## 5 Conclusions

Using surgical pathology after CRT, we developed a machine learning model based on routinely available clinicopathologic variables to estimate pCR in MIBC. WeightedEnsemble_L4 had an AUC of 0.829 in the test cohort. In exploratory analyses, ≥cT3, tumor size >30 mm, concomitant CIS, and multiplicity were associated with lower pCR rates. Independent external validation is required to support future clinical implementation of the model to guide bladder-preservation treatment decisions.

## CRediT authorship contribution statement

**Motohiro Fujiwara:** Writing – original draft, Data curation. **Hajime Tanaka:** Writing – review & editing, Supervision, Conceptualization. **Daisuke Hirahara:** Formal analysis. **Tatsunori Saho:** Supervision. **Eichi Takaya:** Formal analysis. **Shunya Matsumoto:** Data curation. **Kasumi Yoshitomi:** Data curation. **Masaki Kobayashi:** Supervision. **Yuki Nakamura:** Supervision. **Wei Chen:** Writing – review & editing. **Yudai Ishikawa:** Writing – review & editing. **Shohei Fukuda:** Supervision. **Hiroshi Fukushima:** Supervision. **Yuma Waseda:** Writing – review & editing. **Soichiro Yoshida:** Writing – review & editing. **Yasuhisa Fujii:** Project administration.

## Ethics declaration

Written informed consent to take part in the study and to publish the article has been obtained from all participants or their legal representatives. The privacy rights of participants have been observed.

This study was performed in compliance with relevant laws, regulatory frameworks and guidelines where the research took place. This study was approved by the Institute of Science Tokyo. (Approval No. M2019–192)

## Declaration of competing interest

The authors declare that they have no known competing financial interests or personal relationships that could have appeared to influence the work reported in this paper.

## Appendix A Supplementary data

Supplementary material 1

Supplementary material 2 Supplementary Fig. 1 Calibration and exploratory decision curve analyses for WeightedEnsemble_L4 in the test cohort. The calibration slope was 0.856 (95% confidence interval, 0.469–1.639).
