# Supplementary material for: Machine learning models to predict pathological complete response after chemoradiotherapy in muscle-invasive bladder cancer: toward optimized indications for bladder preservation
Source: Clin Transl Radiat Oncol. 2026 Oct 13;62:101320. doi: 10.1016/j.ctro.2026.101320 (PMC13741961; doi:10.1016/j.ctro.2026.101320)

Supplementary Fig. 1A

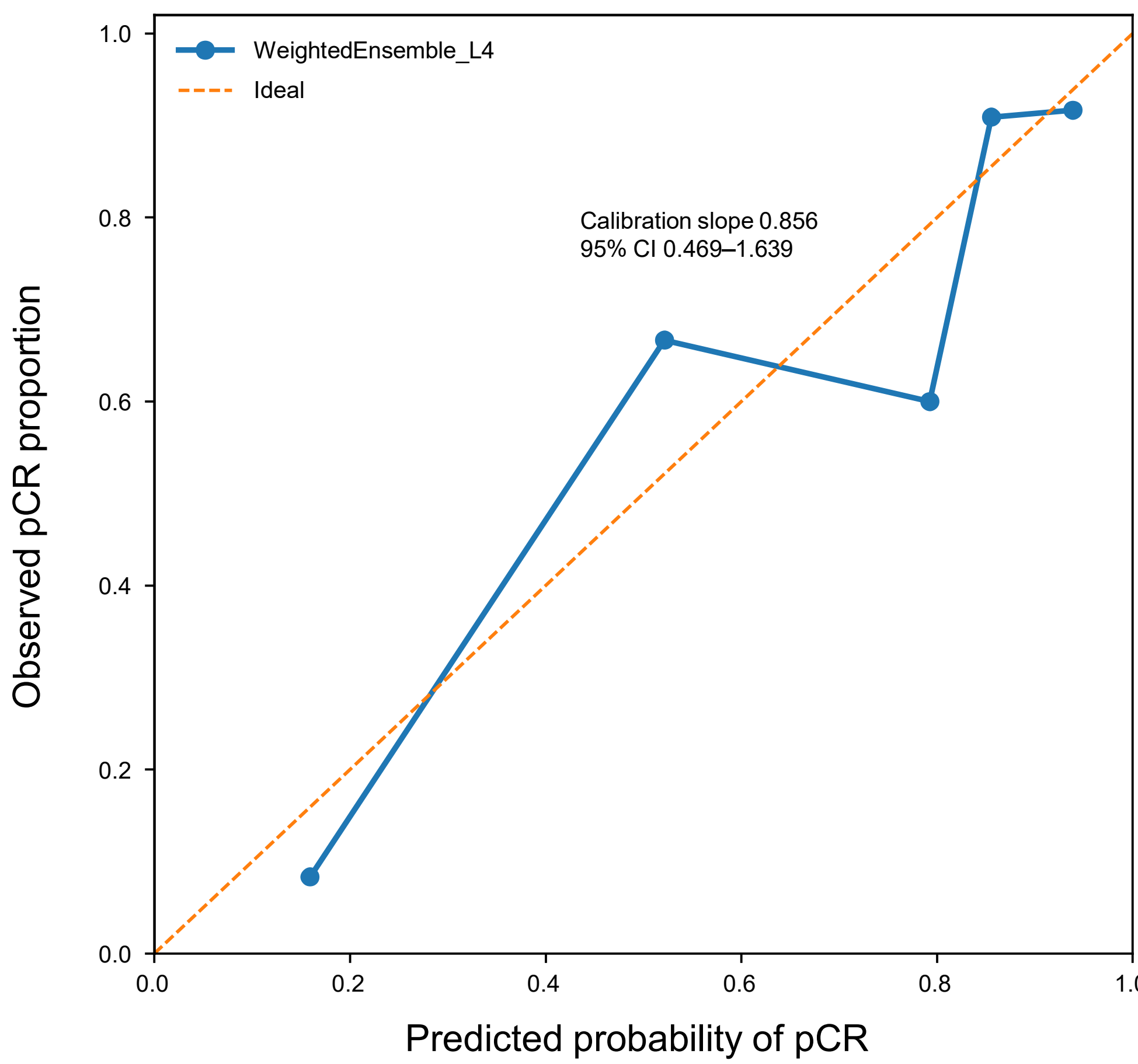

Supplementary Fig. 1B

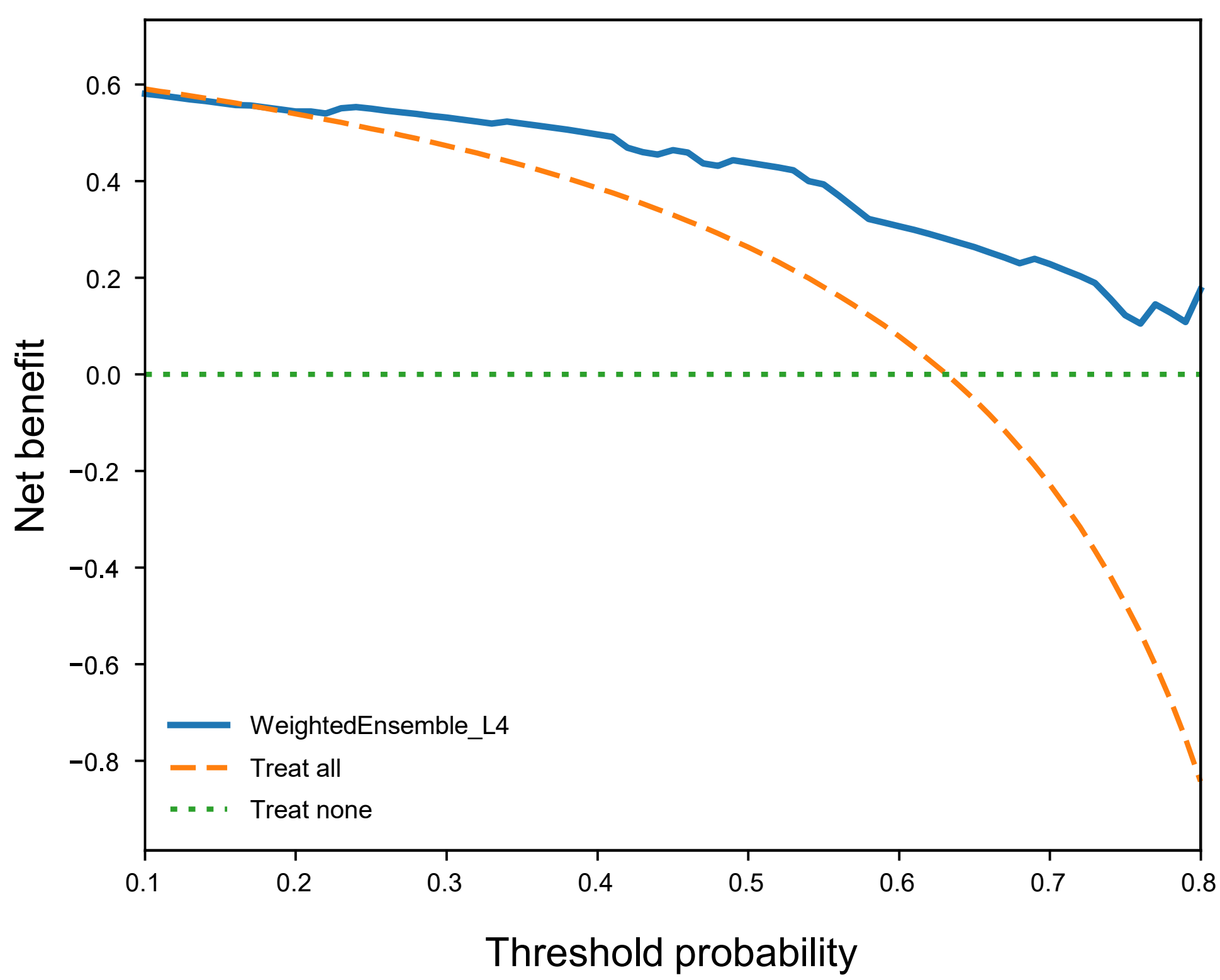

Supplement: Supplementary file 1 [file mmc2.pdf]
