# Supplementary material for: Machine learning models to predict pathological complete response after chemoradiotherapy in muscle-invasive bladder cancer: toward optimized indications for bladder preservation
Source: Clin Transl Radiat Oncol. 2026 Oct 13;62:101320. doi: 10.1016/j.ctro.2026.101320 (PMC13741961; doi:10.1016/j.ctro.2026.101320)
Supplement: Supplementary file 2 [file mmc1.docx]

**Machine learning models to predict pathological complete response after chemoradiotherapy in muscle-invasive bladder cancer: toward optimized indications for bladder preservation**

**Supplementary Materials and Methods**

**1. Overview**

This supplementary document provides additional technical detail on the machine learning workflow used to develop a prediction model for pathological complete response (pCR) after chemoradiotherapy (CRT) in patients with muscle-invasive bladder cancer.

**2. Data partitioning and test cohort**

The dataset included 285 patients. A fixed random split (test_size = 0.20, random_state = 11) assigned 228 patients to the training cohort and 57 to the test cohort. The split was performed without stratification. Model training, internal ranking, and final model selection were completed in the training cohort before test-cohort performance was examined. The test cohort was not used for model training, hyperparameter settings, or final model selection.

**3. Predictors**

Nine pretreatment clinicopathologic variables were used: clinical T stage (≥cT3), maximum tumor diameter (>30 mm), bladder neck involvement, hydronephrosis, multiplicity, concomitant carcinoma in situ, primary histologic grade (G3), variant histology, and progression from prior non-muscle-invasive bladder cancer. Tumor size was the maximum diameter measured on pretreatment computed tomography or magnetic resonance imaging. Primary histologic grade was assessed in the pretreatment transurethral resection specimen with the 1999 WHO classification. Progression from prior non-muscle-invasive bladder cancer was defined as recurrence as muscle-invasive bladder cancer after prior pathologically confirmed non-muscle-invasive bladder cancer had been treated.

**4. Conventional benchmark**

For comparison with a simpler model, we evaluated AutoGluon LinearModel_BAG_L1, a base-level regularized logistic regression model. This model used only the nine original clinical variables and did not use predictions from other models as additional inputs.

**5. AutoGluon model development**

Machine learning used AutoGluon Tabular version 1.3.1 with Python 3.11.13. The prediction target was pCR, and the model-selection metric was area under the receiver operating characteristic curve (AUC). Training used the best_quality preset with a 7,200-second time limit.

We specified 36 model configurations: NeuralNetTorch (5), NeuralNetFastAI (5), XGBoost (5), LightGBM (5), CatBoost (4), random forest (3), Extra Trees (3), linear model (3), and k-nearest-neighbor (3). The three k-nearest-neighbor configurations were skipped because no valid features remained after their preprocessing steps, leaving 33 trainable configurations.

The fitted AutoGluon settings were num_bag_folds = 8, num_bag_sets = 1, and num_stack_levels = 2; dynamic stacking was disabled. The 33 trainable configurations were fitted at three model levels, and three weighted ensembles were generated, yielding 102 candidate models. Bagging and stacking used only the training cohort.

**6. Final model selection and test-cohort evaluation**

Candidate models were ranked by AUC from AutoGluon internal validation in the training cohort. WeightedEnsemble_L4 had the highest training-cohort validation AUC (0.921) and was selected as the final model before test-cohort performance was examined. The final ensemble combined CatBoost_3_BAG_L3 (weight 0.75), LightGBM_BAG_L3 (0.125), and NeuralNetFastAI_5_BAG_L3 (0.125).

The selected final model was then evaluated in the 57-patient test cohort. Discrimination was measured with AUC. Calibration was summarized with a calibration plot and calibration slope. Ninety-five percent confidence intervals for test-cohort AUC and calibration slope were calculated with 2,000 stratified bootstrap samples. Decision curve analysis was retained as an exploratory analysis for WeightedEnsemble_L4. Test-cohort results from unselected candidate models are not presented in the revised Supplementary Materials.

**7. Additional five-fold internal validation**

As an additional internal validation, we repeated model development using five different train/test splits. In each analysis, 228 patients were used for model development and 57 patients were reserved for evaluation, with a similar proportion of pCR and non-pCR cases. The AUCs of WeightedEnsemble_L4 were 0.668, 0.630, 0.766, 0.691, and 0.702, with a mean AUC of 0.691. Results are shown in Supplementary Table 4.

**8. Exploratory predictor ranking**

After final model selection and test-cohort performance assessment, we performed an exploratory permutation analysis in the training cohort. For each predictor, values were shuffled 50 times and the change in AUC was measured. The analysis was performed across eight representative level-3 AutoGluon models, and the mean AUC decrease for each predictor was summarized across models. This analysis was performed after final model selection and was not used for model training or model selection.

**Supplementary Tables**

**Supplementary Table 1. TRIPOD checklist for prediction model development**

| **Section/Topic** | **Item** | **Checklist item** | **Page** |
| --- | --- | --- | --- |
| Title and abstract | 1 | Identify the study as developing and/or validating a multivariable prediction model, the target population, and the outcome to be predicted. | 1-3 |
| Abstract | 2 | Provide a summary of objectives, study design, setting, participants, sample size, predictors, outcome, analysis, results, and conclusions. | 2-3 |
| Background and objectives | 3a | Explain the medical rationale for developing or validating the prediction model, including references to existing models. | 3-4 |
| Background and objectives | 3b | Specify whether the study describes model development, validation, or both. | 3-4 |
| Source of data | 4a | Describe the study design or source of data for the development and validation data sets. | 4 |
| Source of data | 4b | Specify key study dates. | 4 |
| Participants | 5a | Specify the study setting and center. | 4 |
| Participants | 5b | Describe eligibility criteria. | 4-5 |
| Participants | 5c | Give details of treatments received, if relevant. | 5 |
| Outcome | 6a | Define the predicted outcome, including how and when it was assessed. | 5 |
| Outcome | 6b | Report blinding of outcome assessment, if applicable. | N/A |
| Predictors | 7a | Define all predictors, including how and when they were measured. | 5-6 |
| Predictors | 7b | Report blinding of predictor assessment, if applicable. | N/A |
| Sample size | 8 | Explain how the study size was determined. | 4 |
| Missing data | 9 | Describe how missing data were handled. | 7 |
| Analysis methods | 10a | Describe how predictors were handled. | 5-6 |
| Analysis methods | 10b | Specify model types, model-building procedures, and internal validation. | 6; Suppl. pp. 1-3 |
| Analysis methods | 10d | Specify measures used to assess model performance. | 6; Suppl. p. 2 |
| Risk groups | 11 | Describe how risk groups were created, if applicable. | 8-9 |
| Participants | 13a | Describe participant flow and outcome counts. | 4, 7-8 |
| Participants | 13b | Describe participant characteristics and missing data. | 8; Table 1 |
| Model development | 14a | Specify the number of participants and outcome events in each analysis. | 7-8 |
| Model development | 14b | Report unadjusted associations, if done. | 8-9 |
| Model specification | 15a | Present the full model where applicable. | Suppl. p. 2 |
| Model specification | 15b | Explain how to use the model. | N/A |
| Model performance | 16 | Report model performance measures with confidence intervals. | 2, 8; Suppl. pp. 2-3 |
| Limitations | 18 | Discuss study limitations. | 11-12 |
| Interpretation | 19b | Interpret the results considering objectives, limitations, and related evidence. | 9-11 |
| Implications | 20 | Discuss potential clinical use and future research. | 10-11 |
| Supplementary information | 21 | Provide information about supplementary resources. | 6, 8, 18 |
| Funding | 22 | Give the funding source and role of funders. | N/A |

N/A, not applicable; Suppl., Supplementary Materials and Methods.

**Supplementary Table 2. Pathological findings on cystectomy specimens**

|  | **All patients (n = 285)** | **Training cohort (n = 228)** | **Test cohort (n = 57)** | **p value** |
| --- | --- | --- | --- | --- |
| Surgery |  |  |  | 0.554 |
| Partial cystectomy | 148 (52) | 116 (51) | 32 (56) |  |
| Radical cystectomy | 137 (48) | 112 (49) | 25 (44) |  |
| Pathological T stage |  |  |  | 0.773 |
| ypT0 (pCR) | 178 (62) | 142 (62) | 36 (63) |  |
| ypTa–is | 21 (7) | 17 (7) | 4 (7) |  |
| ypT1 | 22 (8) | 16 (7) | 6 (11) |  |
| ypT2–4 | 64 (22) | 53 (23) | 11 (19) |  |

Values are n (%). Abbreviations: pCR, pathological complete response.

**Supplementary Table 3. Candidate models ranked by training-cohort validation AUC**

| **Rank** | **Model** | **Algorithm** | **Level** | **Validation AUC** |
| --- | --- | --- | --- | --- |
| 1 | WeightedEnsemble_L4 | Weighted ensemble | L4 | 0.921 |
| 2 | WeightedEnsemble_L3 | Weighted ensemble | L3 | 0.919 |
| 3 | CatBoost_3_BAG_L3 | CatBoost | L3 | 0.918 |
| 4 | CatBoost_BAG_L3 | CatBoost | L3 | 0.915 |
| 5 | CatBoost_4_BAG_L3 | CatBoost | L3 | 0.914 |
| 6 | LightGBM_BAG_L3 | LightGBM | L3 | 0.913 |
| 7 | CatBoost_2_BAG_L3 | CatBoost | L3 | 0.913 |
| 8 | LightGBM_2_BAG_L3 | LightGBM | L3 | 0.912 |
| 9 | LightGBM_4_BAG_L3 | LightGBM | L3 | 0.912 |
| 10 | LightGBM_5_BAG_L3 | LightGBM | L3 | 0.911 |
| 11 | XGBoost_5_BAG_L3 | XGBoost | L3 | 0.907 |
| 12 | XGBoost_BAG_L3 | XGBoost | L3 | 0.906 |
| 13 | LightGBM_3_BAG_L3 | LightGBM | L3 | 0.905 |
| 14 | XGBoost_4_BAG_L3 | XGBoost | L3 | 0.904 |
| 15 | XGBoost_2_BAG_L3 | XGBoost | L3 | 0.902 |
| 16 | NeuralNetTorch_3_BAG_L3 | PyTorch neural network | L3 | 0.902 |
| 17 | XGBoost_3_BAG_L3 | XGBoost | L3 | 0.900 |
| 18 | ExtraTrees_3_BAG_L3 | Extra Trees | L3 | 0.898 |
| 19 | RandomForest_BAG_L3 | Random forest | L3 | 0.897 |
| 20 | NeuralNetTorch_2_BAG_L3 | PyTorch neural network | L3 | 0.897 |
| 21 | RandomForest_3_BAG_L3 | Random forest | L3 | 0.897 |
| 22 | RandomForest_2_BAG_L3 | Random forest | L3 | 0.896 |
| 23 | ExtraTrees_2_BAG_L3 | Extra Trees | L3 | 0.896 |
| 24 | NeuralNetFastAI_5_BAG_L3 | FastAI neural network | L3 | 0.896 |
| 25 | ExtraTrees_BAG_L3 | Extra Trees | L3 | 0.895 |
| 26 | NeuralNetFastAI_2_BAG_L3 | FastAI neural network | L3 | 0.894 |
| 27 | NeuralNetFastAI_3_BAG_L2 | FastAI neural network | L2 | 0.894 |
| 28 | NeuralNetFastAI_4_BAG_L3 | FastAI neural network | L3 | 0.894 |
| 29 | NeuralNetFastAI_BAG_L3 | FastAI neural network | L3 | 0.892 |
| 30 | NeuralNetTorch_BAG_L3 | PyTorch neural network | L3 | 0.890 |
| 31 | NeuralNetTorch_5_BAG_L3 | PyTorch neural network | L3 | 0.890 |
| 32 | NeuralNetFastAI_4_BAG_L2 | FastAI neural network | L2 | 0.889 |
| 33 | NeuralNetTorch_4_BAG_L3 | PyTorch neural network | L3 | 0.889 |
| 34 | LinearModel_BAG_L3 | Regularized logistic regression | L3 | 0.886 |
| 35 | NeuralNetFastAI_3_BAG_L3 | FastAI neural network | L3 | 0.885 |
| 36 | NeuralNetFastAI_2_BAG_L2 | FastAI neural network | L2 | 0.882 |
| 37 | NeuralNetFastAI_5_BAG_L2 | FastAI neural network | L2 | 0.880 |
| 38 | LinearModel_2_BAG_L3 | Regularized logistic regression | L3 | 0.877 |
| 39 | LinearModel_3_BAG_L3 | Regularized logistic regression | L3 | 0.873 |
| 40 | NeuralNetTorch_4_BAG_L2 | PyTorch neural network | L2 | 0.860 |
| 41 | NeuralNetFastAI_BAG_L2 | FastAI neural network | L2 | 0.859 |
| 42 | CatBoost_BAG_L2 | CatBoost | L2 | 0.853 |
| 43 | NeuralNetTorch_BAG_L2 | PyTorch neural network | L2 | 0.849 |
| 44 | LinearModel_3_BAG_L2 | Regularized logistic regression | L2 | 0.847 |
| 45 | NeuralNetTorch_5_BAG_L2 | PyTorch neural network | L2 | 0.842 |
| 46 | CatBoost_4_BAG_L2 | CatBoost | L2 | 0.836 |
| 47 | CatBoost_2_BAG_L2 | CatBoost | L2 | 0.834 |
| 48 | NeuralNetTorch_2_BAG_L2 | PyTorch neural network | L2 | 0.833 |
| 49 | ExtraTrees_BAG_L2 | Extra Trees | L2 | 0.830 |
| 50 | RandomForest_BAG_L2 | Random forest | L2 | 0.830 |
| 51 | CatBoost_3_BAG_L2 | CatBoost | L2 | 0.828 |
| 52 | ExtraTrees_2_BAG_L2 | Extra Trees | L2 | 0.821 |
| 53 | LinearModel_2_BAG_L2 | Regularized logistic regression | L2 | 0.820 |
| 54 | RandomForest_2_BAG_L2 | Random forest | L2 | 0.817 |
| 55 | XGBoost_BAG_L2 | XGBoost | L2 | 0.808 |
| 56 | LightGBM_4_BAG_L2 | LightGBM | L2 | 0.805 |
| 57 | NeuralNetTorch_3_BAG_L2 | PyTorch neural network | L2 | 0.803 |
| 58 | LightGBM_2_BAG_L2 | LightGBM | L2 | 0.802 |
| 59 | LightGBM_5_BAG_L2 | LightGBM | L2 | 0.800 |
| 60 | XGBoost_2_BAG_L2 | XGBoost | L2 | 0.798 |
| 61 | LightGBM_BAG_L2 | LightGBM | L2 | 0.796 |
| 62 | RandomForest_3_BAG_L2 | Random forest | L2 | 0.795 |
| 63 | LightGBM_3_BAG_L2 | LightGBM | L2 | 0.792 |
| 64 | XGBoost_4_BAG_L2 | XGBoost | L2 | 0.792 |
| 65 | XGBoost_5_BAG_L2 | XGBoost | L2 | 0.791 |
| 66 | ExtraTrees_3_BAG_L2 | Extra Trees | L2 | 0.786 |
| 67 | XGBoost_3_BAG_L2 | XGBoost | L2 | 0.780 |
| 68 | WeightedEnsemble_L2 | Weighted ensemble | L2 | 0.771 |
| 69 | NeuralNetFastAI_4_BAG_L1 | FastAI neural network | L1 | 0.763 |
| 70 | NeuralNetFastAI_3_BAG_L1 | FastAI neural network | L1 | 0.759 |
| 71 | NeuralNetFastAI_2_BAG_L1 | FastAI neural network | L1 | 0.758 |
| 72 | NeuralNetFastAI_5_BAG_L1 | FastAI neural network | L1 | 0.757 |
| 73 | LinearModel_BAG_L2 | Regularized logistic regression | L2 | 0.755 |
| 74 | NeuralNetFastAI_BAG_L1 | FastAI neural network | L1 | 0.749 |
| 75 | XGBoost_3_BAG_L1 | XGBoost | L1 | 0.727 |
| 76 | CatBoost_3_BAG_L1 | CatBoost | L1 | 0.723 |
| 77 | NeuralNetTorch_3_BAG_L1 | PyTorch neural network | L1 | 0.722 |
| 78 | NeuralNetTorch_4_BAG_L1 | PyTorch neural network | L1 | 0.719 |
| 79 | XGBoost_5_BAG_L1 | XGBoost | L1 | 0.710 |
| 80 | CatBoost_4_BAG_L1 | CatBoost | L1 | 0.708 |
| 81 | XGBoost_2_BAG_L1 | XGBoost | L1 | 0.707 |
| 82 | XGBoost_BAG_L1 | XGBoost | L1 | 0.706 |
| 83 | CatBoost_BAG_L1 | CatBoost | L1 | 0.706 |
| 84 | LightGBM_2_BAG_L1 | LightGBM | L1 | 0.705 |
| 85 | CatBoost_2_BAG_L1 | CatBoost | L1 | 0.705 |
| 86 | LightGBM_4_BAG_L1 | LightGBM | L1 | 0.704 |
| 87 | LightGBM_5_BAG_L1 | LightGBM | L1 | 0.704 |
| 88 | XGBoost_4_BAG_L1 | XGBoost | L1 | 0.703 |
| 89 | NeuralNetTorch_2_BAG_L1 | PyTorch neural network | L1 | 0.702 |
| 90 | LightGBM_BAG_L1 | LightGBM | L1 | 0.701 |
| 91 | LightGBM_3_BAG_L1 | LightGBM | L1 | 0.698 |
| 92 | NeuralNetTorch_5_BAG_L1 | PyTorch neural network | L1 | 0.693 |
| 93 | ExtraTrees_3_BAG_L1 | Extra Trees | L1 | 0.692 |
| 94 | RandomForest_3_BAG_L1 | Random forest | L1 | 0.691 |
| 95 | LinearModel_BAG_L1 | Regularized logistic regression | L1 | 0.683 |
| 96 | RandomForest_2_BAG_L1 | Random forest | L1 | 0.680 |
| 97 | LinearModel_3_BAG_L1 | Regularized logistic regression | L1 | 0.680 |
| 98 | LinearModel_2_BAG_L1 | Regularized logistic regression | L1 | 0.680 |
| 99 | NeuralNetTorch_BAG_L1 | PyTorch neural network | L1 | 0.679 |
| 100 | ExtraTrees_2_BAG_L1 | Extra Trees | L1 | 0.676 |
| 101 | ExtraTrees_BAG_L1 | Extra Trees | L1 | 0.660 |
| 102 | RandomForest_BAG_L1 | Random forest | L1 | 0.659 |

Models are ranked only by validation AUC from the training cohort. Test-cohort results for unselected models are not shown. WeightedEnsemble_L4 was selected before test-cohort performance was examined.

**Supplementary Table 4. Additional five-fold internal validation of WeightedEnsemble_L4**

| **Fold** | **Training n** | **Held-out n** | **Models trained** | **WeightedEnsemble_L4 internal validation AUC** | **WeightedEnsemble_L4 held-out AUC** |
| --- | --- | --- | --- | --- | --- |
| 1 | 228 | 57 | 99 | 0.965 | 0.668 |
| 2 | 228 | 57 | 102 | 0.939 | 0.630 |
| 3 | 228 | 57 | 101 | 0.972 | 0.766 |
| 4 | 228 | 57 | 102 | 0.939 | 0.691 |
| 5 | 228 | 57 | 100 | 0.901 | 0.702 |
| Mean | 228 | 57 | — | 0.943 | 0.691 |

In each fold, the full AutoGluon procedure was rebuilt using 228 patients, and the WeightedEnsemble_L4 generated in that fold was evaluated in 57 held-out patients. The held-out patients were not used for model development. The mean held-out AUC was 0.691 (range, 0.630–0.766).
